# Bilateral Neck of Femur Fracture in a Child With Underlying Vitamin D Deficiency: A Case Report on Management and 10-Year Follow-Up

**DOI:** 10.7759/cureus.22953

**Published:** 2022-03-08

**Authors:** Anand K Gupta, Amit Narang, Sumit Gupta, Rajesh K Kanojia

**Affiliations:** 1 Orthopaedics, Lady Hardinge Medical College, New Delhi, IND

**Keywords:** bilateral femoral neck stress fracture, vitamin-d deficiency, valgus osteotomy, non-union of fracture neck of femur, fracture neck of femur in children

## Abstract

Bilateral femoral neck fracture is a rare entity in the pediatric age group. These types of fractures occur mostly due to high-velocity trauma. We report the surgical outcome with approximately 10 years of follow-up in a 10-year-old child presenting with bilateral femoral neck fracture after trivial trauma and underlying nutritional vitamin D deficiency. A 10-year-old female child with bilateral neglected fracture neck of femur was managed with a primary valgus osteotomy done on the left side and closed reduction and screw fixation with fibular grafting done on the right side. Later on, as the right side fracture progressed to non-union, it was converted to valgus osteotomy fixed with an external fixator. The patient had a good functional outcome at 10 years of follow-up with no difficulty in her day-to-day activities. Pathological bilateral fracture neck of femur is rare in children and it is often mismanaged due to a delayed diagnosis. It can have potentially dangerous complications with a grave outcome affecting the rest of the life of the child. Hence it is important to know about such rare presentations so that they can be adequately addressed early on, thereby minimizing the risk of complications like non-union and avascular necrosis.

## Introduction

Fracture neck of femur in children constitutes less than 1% of all pediatric trauma cases. Simultaneous bilateral femoral neck fractures in children are even rarer and most cases reported are either due to fall from height or due to high-velocity trauma [1]. We report our experience of bilateral femoral neck fracture in a child attributed to nutritional vitamin D deficiency. The patient was treated surgically with valgus subtrochanteric osteotomy with a good functional outcome. Possible complications of these fractures in children include avascular necrosis, non-union, coxa vara, physeal growth arrest, and limb length discrepancy. There are higher chances of these complications occurring in cases of pathological fractures due to poor bone quality, delayed presentation to the hospital, and a delay in the appropriate treatment due to misdiagnosis [2].

## Case presentation

**Figure 1:**
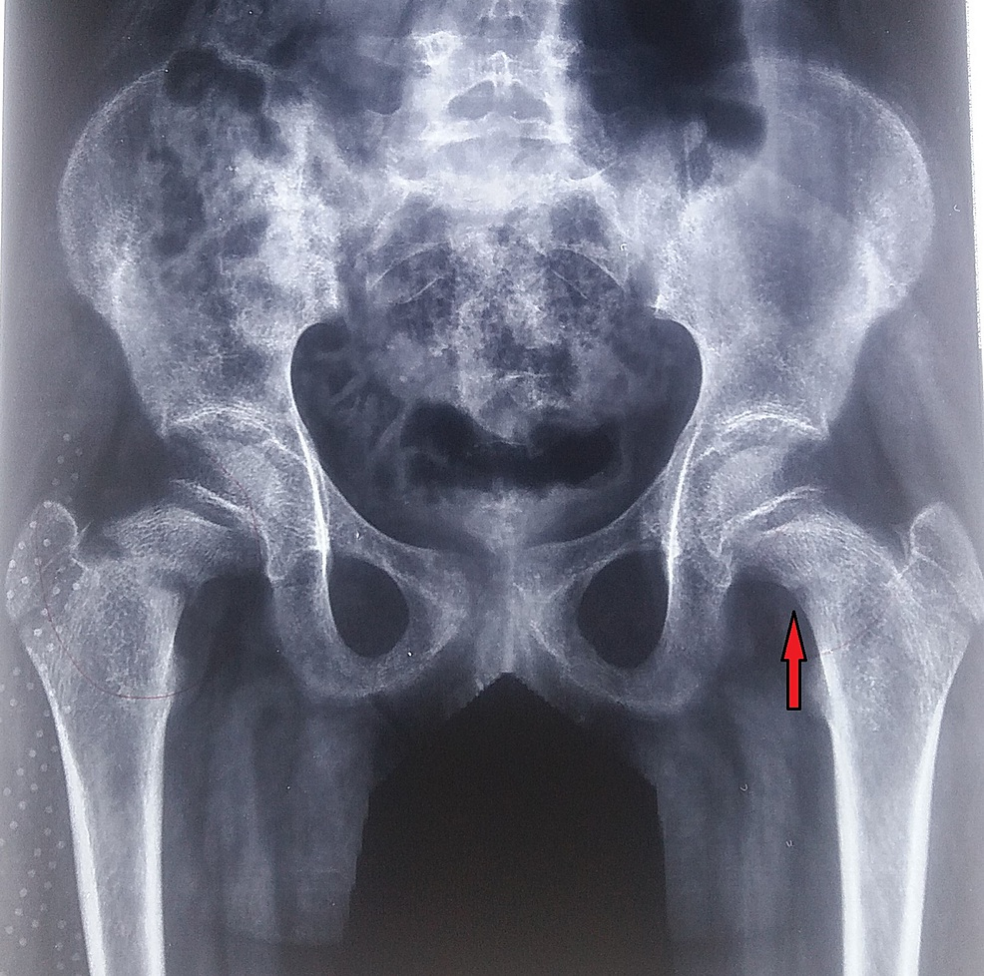
Anteroposterior radiograph of the pelvis and both hips showing bilateral fracture neck of femur (left side marked with red arrow).

A 10-year-old female child presented to us in June 2010 with complaints of pain in the bilateral hip region for three months, and inability to walk for one month with a history of fall from bed one month back. Due to the remote location of her village, there was a delay in seeking institutional treatment. Apart from trivial trauma, her medical history was unremarkable. On clinical examination, both the lower limbs were in external rotation, bilateral anterior hip joint tenderness was present and, the patient was not able to perform an active SLR (straight leg raising) test. Radiographs of both hips revealed Delbet type II fracture neck of the femur on the right side and type III fracture on the left side with proximal migration of both the greater trochanters (Figure 1). There were also signs of healing fracture of the superior and inferior pubic rami on the right side. Laboratory tests showed a decrease in serum vitamin D levels (8.5 ng/ml) and increased alkaline phosphatase levels. Prior to the fractures, the child was asymptomatic, and without notable medical history. Nutritional vitamin D deficiency and subsequent bilateral insufficiency fracture of the femoral neck was the final hypothesis.

**Figure 2:**
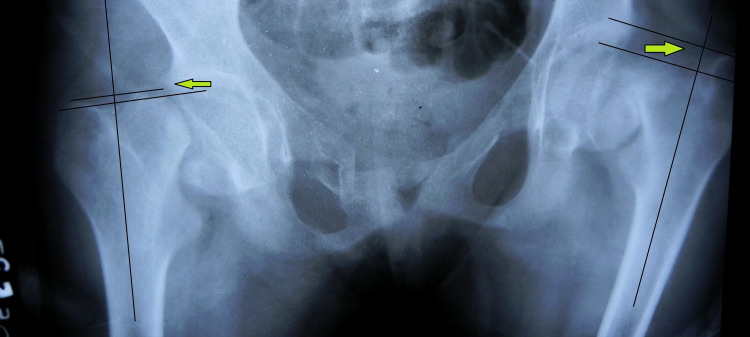
Anteroposterior radiograph of the pelvis and both hips showing bilateral fracture neck of femur with negative articular-trochanteric distance (green arrow).

As the articular-trochanteric distance was negative on both sides (left > right), bilateral skeletal traction was applied (Figure 2).

**Figure 3:**
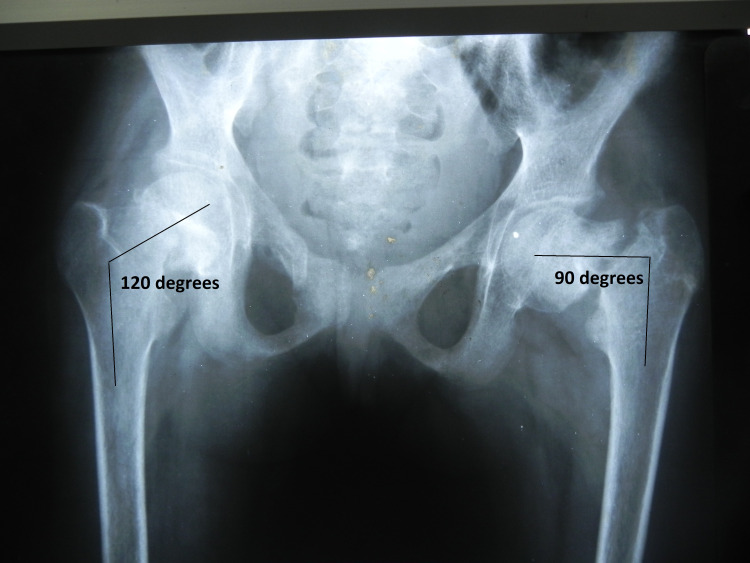
Radiograph after two weeks of skeletal traction showed neck shaft angle was 120° on the right side and 90° on the left side.

She was given an intramuscular injection of 600000 IU of vitamin D followed by a maintenance oral daily dose of 400 IU along with oral calcium. After two weeks of skeletal traction, the neck shaft angle was 120° on the right side and 90° on the left side (Figure 3).

**Figure 4:**
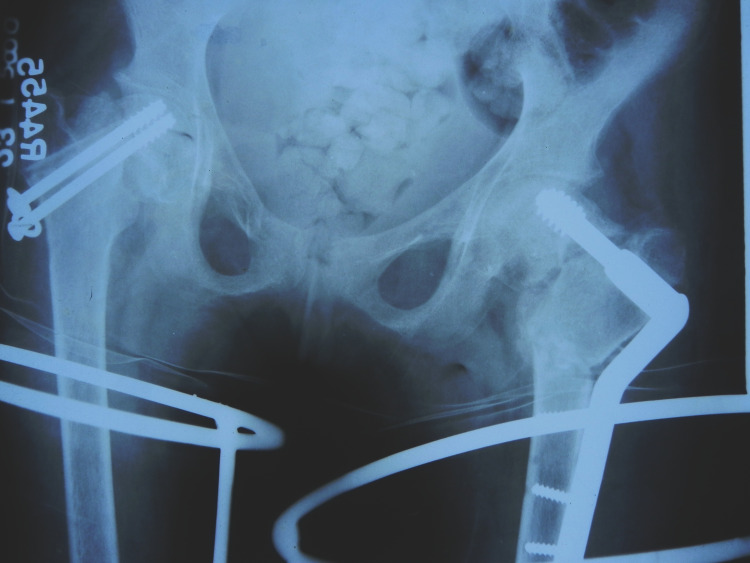
Anteroposterior radiograph of the bilateral hip showing cannulated cancellous screw fixation in the right hip and valgus osteotomy in the left hip.

So, closed reduction and internal fixation with cannulated cancellous screws (CCS) along with non-vascularized fibula augmentation were planned on the right side and a primary femoral valgus osteotomy was planned on the left side (Figure 4).

**Figure 5:**
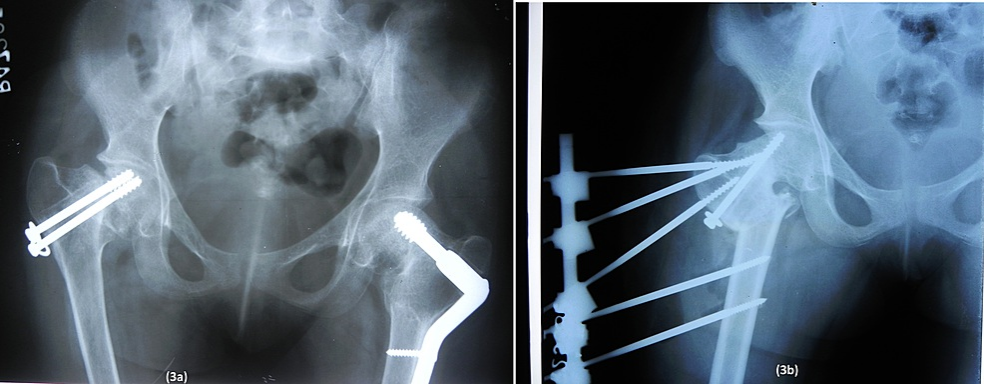
Five-month follow-up radiograph showing non union of the right side. Right side was further revised with valgus osteotomy; osteotomy site was fixed with external fixator.

At five-month follow-up, it was realized that the fracture on the right side was going into non-union while there was union on the left side. The right-sided fracture was revised with valgus osteotomy which was fixed by using an external fixator device. We opted to fix the valgus osteotomy with an external fixator as we were afraid that lag screw insertion might damage the fibula graft which was used in the previous surgery. One CCS screw of previous surgery was left in situ to hold the two fracture fragments together (Figure 5).

The patient was kept non-weight bearing on the right side for a period of six weeks. Gradually at three-month follow-up, fracture of the right hip was also united. Implant removal of both sides was done after complete fracture union. At present, she is 20 years old and can do all her regular activities without any difficulty.

**Figure 6:**
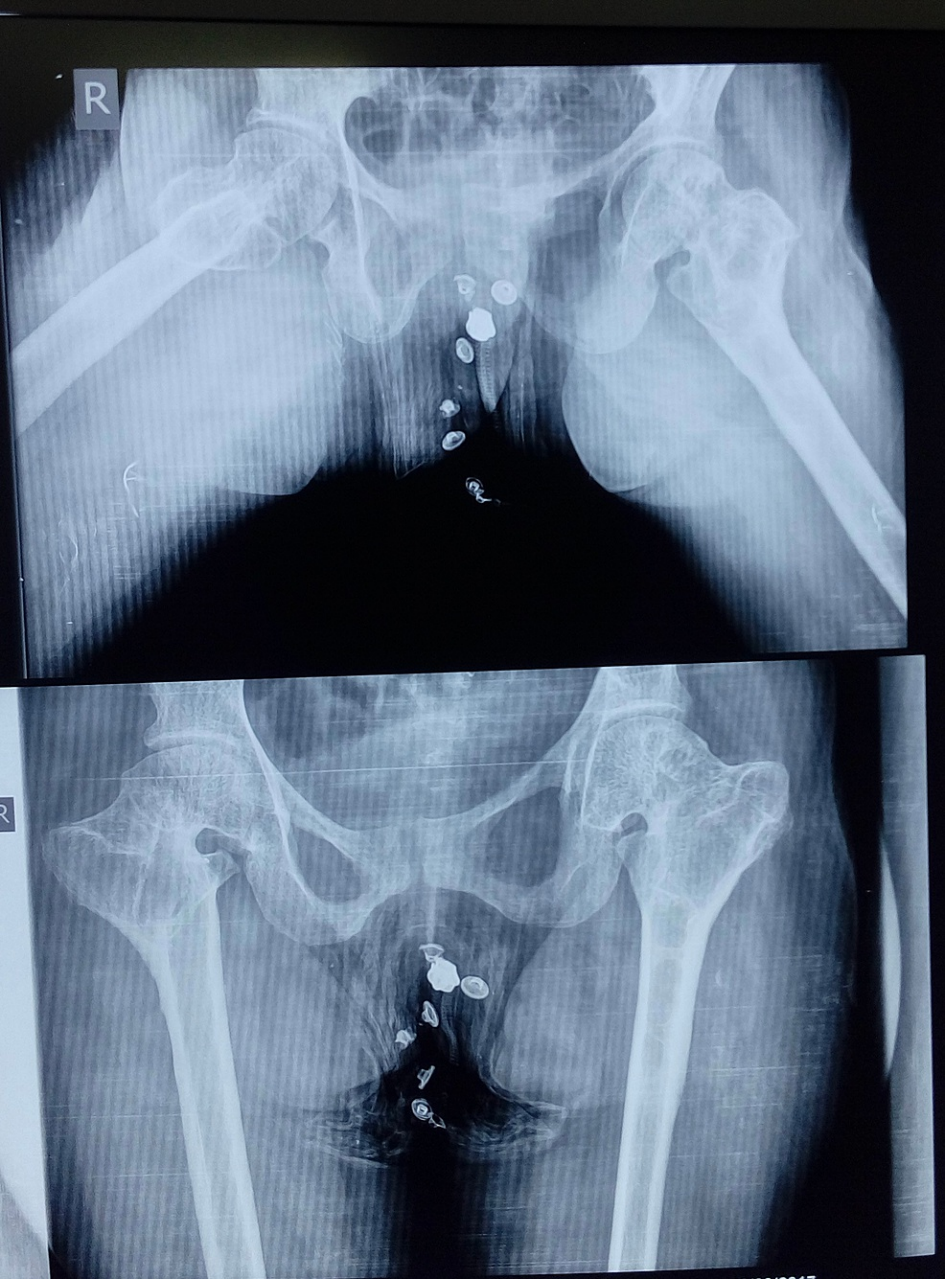
Anteroposterior and lateral radiograph of the bilateral hip at 10-year follow-up.

Recent follow-up radiograph shows that there is a shortening of the femoral neck on both sides and bilateral lesser trochanters are proximal to the ischial tuberosity. On the lateral radiograph, it was observed that the right-sided femoral head was not as spherical as the left one (Figure 6).

**Figure 7:**
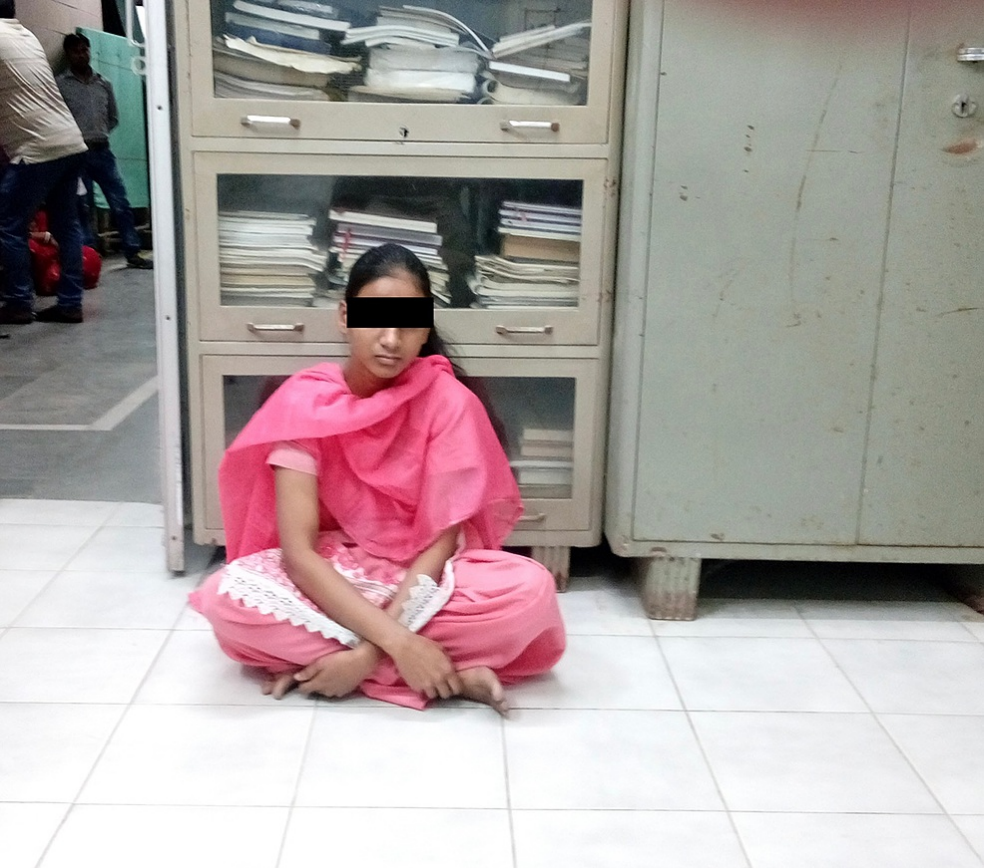
Clinical photograph of the patient at 10-year follow-up.

This may result in early osteoarthritis on the right side in the near future. However, until now, there is no sign of avascular necrosis. The patient has an almost normal range of motion, no limb length discrepancy, no pain, and no abnormality in gait. The patient can sit cross-legged and can squat. Her bilateral Harris hip score was 94.6 (Figure 7).

## Discussion

Simultaneous bilateral fracture neck of femur due to trivial trauma has been commonly reported in old age and pregnant females due to either osteomalacia or osteoporosis. These types of fractures are also reported in patients suffering from renal osteodystrophy, primary hyperparathyroidism, celiac disease, and malignancy. Corticosteroid, anti-epileptic, antacid drugs, and pelvic irradiation are other rare causes [3,4]. A bilateral replacement has been considered as a treatment of choice in the adult patient by most of the authors [5].

Management of bilateral pathological fracture neck of femur in children is challenging due to its associated complications. As these fractures are low-velocity fractures, un-displaced fractures can be managed conservatively. But in displaced fracture, surgical fixation is the treatment of choice. Every attempt should be made to preserve the femoral head, as in children, remodeling occurs till skeletal maturity. Subtrochanteric valgus osteotomy and fibular grafting, both are described methods for old fracture neck of the femur [6]. Neto et al. have reported fair results with angle blade fixation of valgus subtrochanteric osteotomy in non-union fracture neck of femur in children [7]. We chose a double-angle dynamic condylar screw implant due to the relative ease in changing the guide wire placement in the desired portion of the femoral head. Few authors have used external fixator devices to fix subtrochanteric osteotomies in the pediatric age group. Though the application of an external fixator in subtrochanteric osteotomy is technically demanding, it avoids another major operation to remove it [8].

Evaluation of the functional outcome of valgus osteotomy in our patient in terms of pain, joint range of motion, activity level, and radiographic changes as per Ratliff’s concepts reveals a good result even after 10 years of long-term follow-up [9].

Many times, orthopedic surgeons face a diagnostic dilemma while dealing with low-velocity fractures in patients with vitamin D deficiency where all other investigations are normal as subclinical deficiency of vitamin D is very common in the adolescent age group and these children may not present with the classical sign of hypercalcemia. Low dietary intake, poor sunlight in overcrowded localities, high atmospheric pollution filtering ultraviolet rays, and a diet rich in phytates (rice and wheat) are common causes of vitamin D deficiency in the Indian population [10]. It is recommended that any adolescent child presenting with hip pain or abnormal gait should be thoroughly investigated to rule out pathological fracture and its underlying cause. An early diagnosis of osteoarticular pathology can prevent disastrous complications; whereas, in delayed cases presenting with upward migrating greater trochanter, a subtrochanteric valgus osteotomy can be considered as a good surgical option.

## Conclusions

Subclinical vitamin D deficiency should be kept in mind while treating cases of bilateral neck of femur fracture due to trivial trauma. An attempt to diagnose these fractures early can save the patient from disastrous complications; preserving the femoral head is of utmost importance in the pediatric age group. In cases of neglected fractures and varus deformity and/or nonunion, a valgus subtrochanteric osteotomy should be considered as a valuable alternative.
